# Blister formation during graphite surface oxidation by Hummers’ method

**DOI:** 10.3762/bjnano.9.40

**Published:** 2018-02-02

**Authors:** Olga V Sinitsyna, Georgy B Meshkov, Anastasija V Grigorieva, Alexander A Antonov, Inna G Grigorieva, Igor V Yaminsky

**Affiliations:** 1 Laboratory for Physical Chemistry of Polymers, A. N. Nesmeyanov Institute of Organoelement Compounds of Russian Academy of Sciences, Vavilova St. 28, Moscow 119991, Russia; 2 Physics Department, Lomonosov Moscow State University, Leninskie Gory, Moscow 119991, Russia; 3 Department of Materials Science, Lomonosov Moscow State University, Leninskie Gory, Moscow 119991, Russia; 4 Optigraph GmbH, Rudower Chaussee 29, 12489 Berlin, Germany

**Keywords:** atomic force microscopy (AFM), graphene, graphite intercalation compounds (GICs), graphite oxide (GO), highly annealed pyrolythic graphite (HAPG)

## Abstract

Graphite oxide has a complex structure that can be modified in many ways to obtain materials for a wide range of applications. It is known that the graphite precursor has an important role in the synthesis of graphite oxide. In the present study, the basal-plane surface of highly annealed pyrolythic graphite (HAPG) was oxidized by Hummers’ method and investigated by Raman spectroscopy and atomic force microscopy. HAPG was used as a graphite precursor because its surface after cleavage contains well-ordered millimeter-sized regions. The treatment resulted in graphite intercalation by sulfuric acid and blister formation all over the surface. Surprisingly, the destruction of the sp^2^-lattice was not detected in the ordered regions. We suggest that the reagent diffusion under the basal plane surface occurred through the cleavage steps and dislocations with the Burgers vector parallel to the *c*-axis in graphite.

## Introduction

Graphite oxide (GO) and its single-layer derivative, graphene oxide, are of great importance due to their potential applications as a part of supercapacitors, lithium-ion batteries, catalysts, systems for water pollution treatment, nonlinear optical devices and sensors [1–4]. One of the most important applications of graphene oxide is the synthesis of reduced graphene oxide, which exhibits properties similar to graphene [4–5].

The formation of GO involves the intercalation of molecules and ions between the carbon layers, the sp^2^-lattice charging and oxidation [6]. The understanding of these processes is crucial for the reproducible synthesis of GO with desired structure and properties. The larger graphite flakes are used to produce GO, the more important role of the basal-plane surface in the transport of reagents inside the crystals. Eklund et al. [7] noted that sulfuric acid intercalates highly oriented pyrolythic graphite (HOPG) through the basal-plane surface, where the penetration sites are probably grain boundaries, microcracks and atomic steps on the surface.

The present work is devoted to the evaluation of the topography changes during the initial stages of the graphite oxidation in order to clarify the role of the basal-plane surface in the diffusion of reagents. We chose the classical method of GO synthesis proposed by Hummers and Offeman, in which graphite is treated with a mixture of concentrated sulfuric acid, sodium nitrate, and potassium permanganate and then washed with water [8].

Traditionally, HOPG is used as a model material to study the physical and chemical processes occurring on graphite surfaces [9]. HOPG consists of oriented crystallites with almost parallel *c*-axes. The inclination angle between the crystallites is characterized by a mosaic spread, which is between 0.1° and 3° for HOPG [10]. In this paper, we used a new material, highly annealed pyrolythic graphite (HAPG). The mosaic spread of a flat HAPG film can be less than 0.1° [11]. The HAPG surface after cleavage contains well-ordered millimeter-sized regions with a low number of defects, which makes this material convenient for studying the chemistry of the basal-plane surface.

## Results and Discussion

### Treatment of the HAPG surface with the oxidation mixture

After applying the oxidation mixture to the HAPG surface, all the liquid spread over the surface and was absorbed by the graphite during the first minute. The surface became iridescent. Then we did not observe any changes in the state of the surface for 30 minutes. After washing the samples with water and hydrogen peroxide solution, the surface became gray and matte, which indicated a significant change in the surface roughness.

### Raman spectroscopy of the HAPG surface before and after the treatment

**Figure 1:**
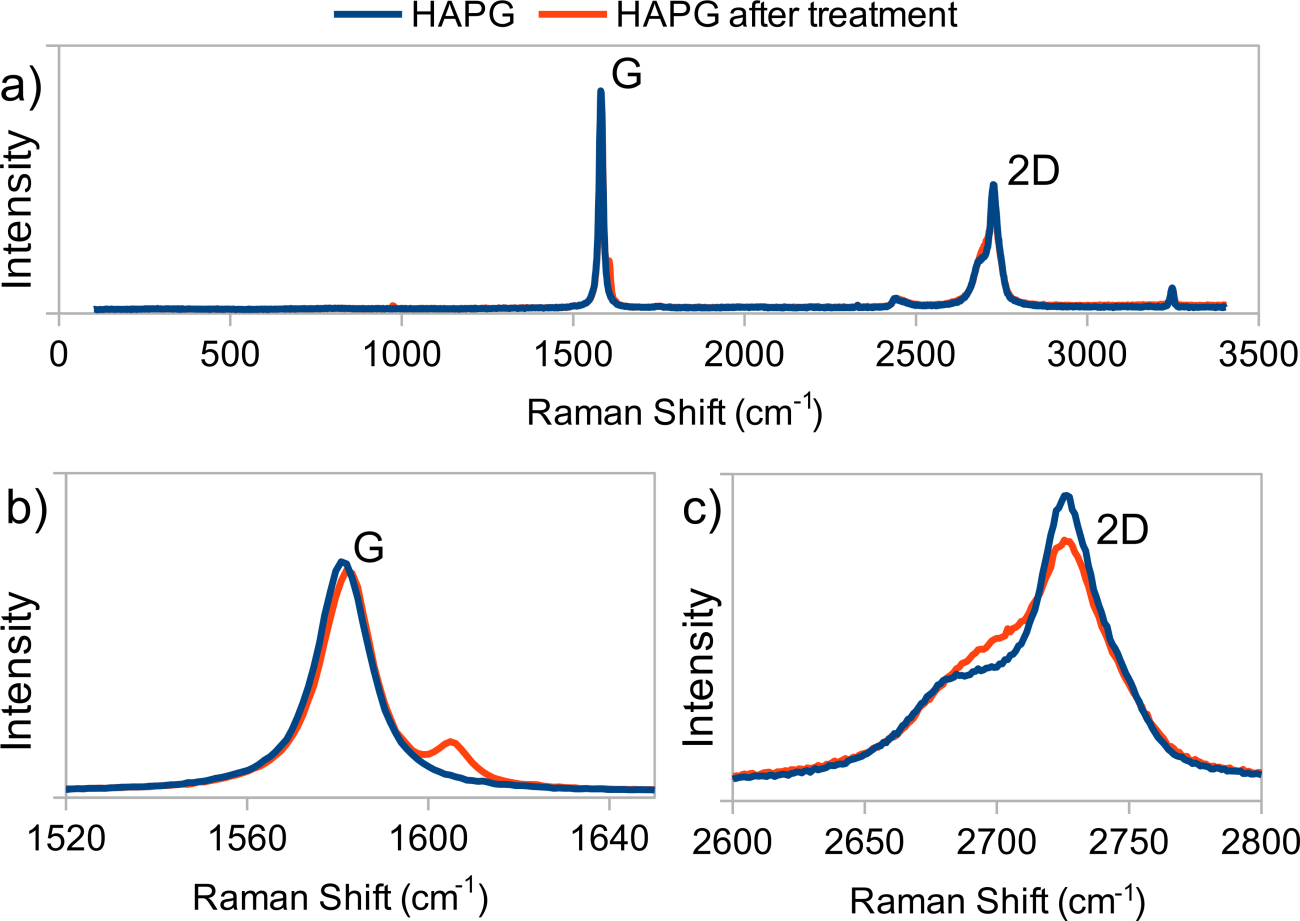
a) Raman spectra of the highly annealed pyrolythic graphite (HAPG) before and after the treatment. G and 2D peaks are shown with higher resolution in b) and c), respectively.

Raman spectra, recorded from the ordered regions on the HAPG surface before and after the treatment, are shown in Figure 1. The absence of the D-peak around ≈1350 cm^−1^ indicates a low content of defects in the carbon layers. Thus, the Raman spectra do not confirm the partial oxidation of the carbon layers and the formation of GO in the ordered regions. It is likely that long-term oxidation is required for the destruction of the carbon layers. According to Dimiev and Tour [6], the GO formation takes hours or days, depending on the type of the graphite precursor.

The appearance of a doublet around 1600 cm^−1^ may indicate the presence of a product of incomplete decomposition of a graphite intercalation compound (GIC) with sulfuric acid. The low-frequency and high-frequency components correspond to the boundary carbon layers with H_2_SO_4_ and the inner layers, respectively [7]. Residual high-stage GICs were also found by X-ray powder diffraction analysis in the products of the graphite bisulfate hydrolysis [12].

The change of the shape of the 2D-peak at ≈2700 cm^−1^ (specifically, an increase in the intensity of the low-frequency part) may be explained by graphene exfoliation from the surface of HAPG [5,13–14].

### Microscopic characterization of the surface changes

#### Microstructure of the HAPG surface before the treatment

**Figure 2:**
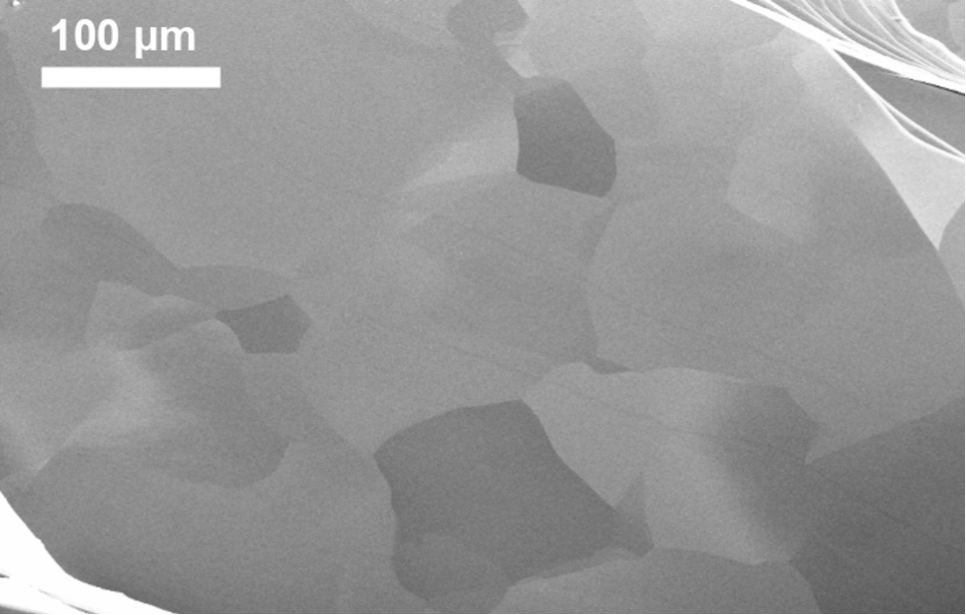
SEM image of the highly annealed pyrolythic graphite (HAPG) surface after cleavage. The sample is tilted by 45° relative to the electron beam to visualize the mosaic structure.

The mosaic structure of the HAPG was examined by scanning electron microscopy (SEM). The HAPG surface after cleavage is shown in Figure 2. The size of the crystallites in the basal plane range from 10 to 200 μm, while for comparison, they are about 10 μm for HOPG with the mosaic spread of 0.8°. The bunches of large cleavage steps are visible in the lower left and the upper right corners of Figure 2.

Steps (linear defects) are the main features in the images obtained by atomic force microscopy (AFM). We distinguish two types of steps: cleavage steps and the lines of edge dislocations with Burgers vector perpendicular to the basal plane. In the second case, atomic steps are located mainly below the surface. The deeper an atomic step lies, the smoother the relief on the surface. In our AFM experiments, we observed surface height changes for the dislocation lines located at a depth of up to 4 nm. The edge dislocations originate in the form of loops during the synthesis of the HAPG. The cleavage steps are often straight and form along the cleavage direction. The number of cleavage steps is probably related to the presence of defects binding the carbon layers together.

**Figure 3:**
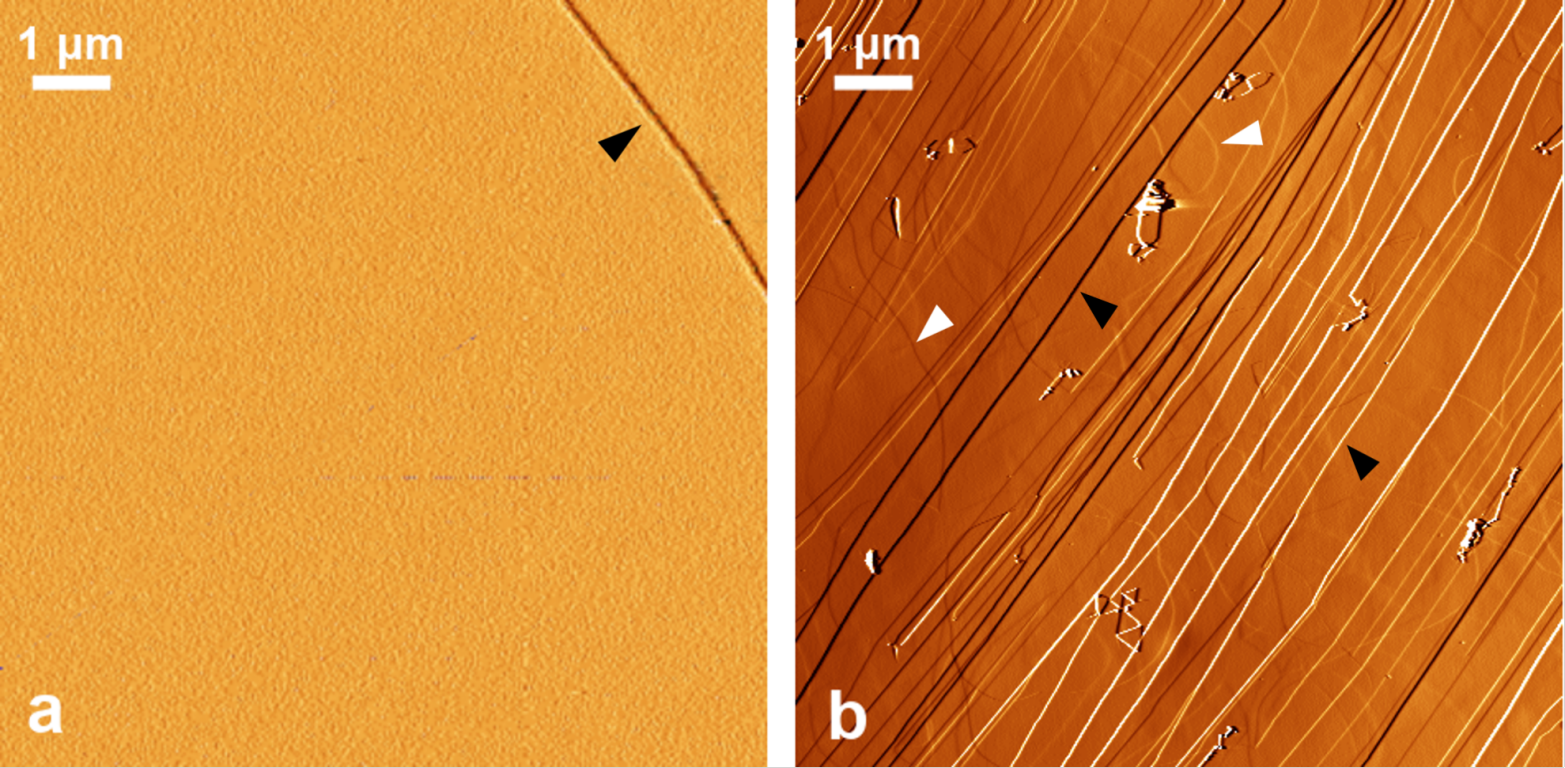
AFM images of highly annealed pyrolythic graphite (HAPG) (a) and highly oriented pyrolythic graphite (HOPG) (b) surfaces after cleavage. A highlight filter was used to enhance the contrast of individual steps. Cleavage steps and lines of the edge dislocations are marked by the black and white arrows, respectively.

The HAPG surface after cleavage contains significantly less defects in the ordered areas than HOPG with mosaic spread of 0.8° (Figure 3a,b). Thus, the length of the cleavage steps per square micrometer in HAPG is 0.2–0.3 μm^−1^, and the length of the dislocation lines per square micrometer is about 0.2 μm^−1^. The lengths of the cleavage steps and the dislocation lines for HOPG are 1–3 μm^−1^ and about 1 μm^−1^, respectively. The height of the cleavage steps is usually less than 1 nm (1–3 carbon layers) for both types of graphite.

Sometimes a cleavage step ends in the center of an atomic terrace, indicating the location of a screw dislocation with the Burgers vector perpendicular to the basal plane. The screw dislocations are rarely observed in AFM images of HAPG. We believe that their length does not exceed several nanometers, since an edge dislocation starting at the site of a screw dislocation is always seen (Figure S1, Supporting Information File 1). As mentioned above, we do not observe the deep-seated edge dislocation in AFM images.

#### AFM examination of the HAPG surface after the treatment

**Figure 4:**
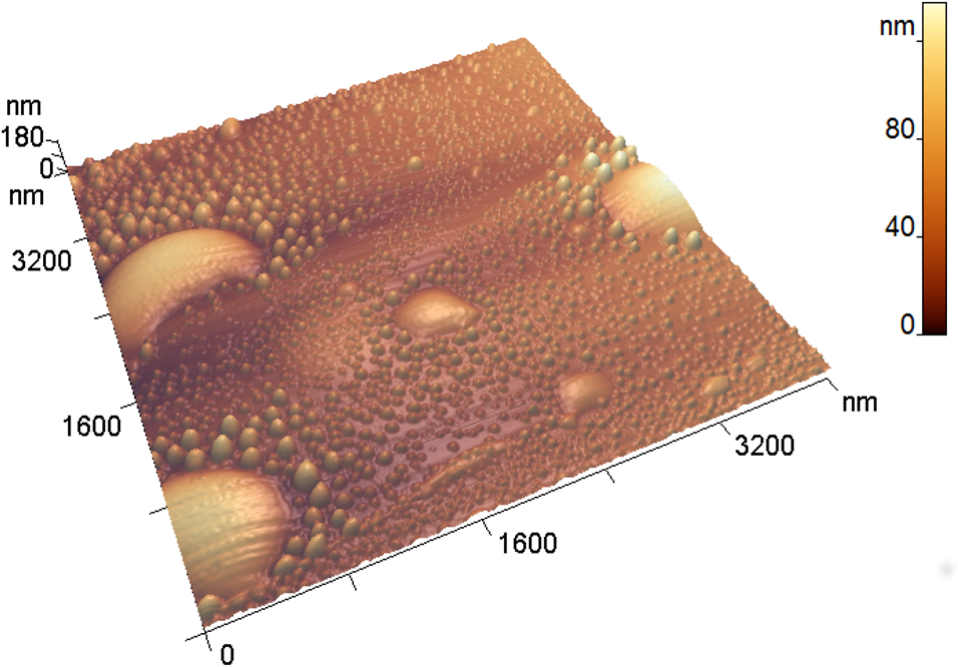
AFM 3D-image of blisters on the highly annealed pyrolythic graphite (HAPG) surface.

The AFM study after the treatment showed that the HAPG surface was covered with blisters (Figure 4). The blisters are unevenly arranged over the surface and have a wide size distribution. We found blisters with a diameter from 14 nm to 1430 nm and a height from 0.5 nm to 187 nm. The root mean square roughness measured in images with an area of 100 μm^2^ increased from 1–2 nm to ≈100 nm. The formation of blisters occurs if the HAPG interacts with the oxidation mixture just for a few minutes, which is confirmed by the results of an experiment in which the exposure time was reduced to 3 min. A typical image of the blisters is shown in Figure S2, Supporting Information File 1.

Similar structures were previously observed on HOPG anodes at the initial stages of electrochemical oxidation in the presence of electrolytes capable of intercalating graphite (i.e., aqueous solutions of KNO_3_ [15], LiClO_4_, (NH_4_)_2_SO_4_, HNO_3_, H_2_SO_4_) [16–17]. The electrochemical oxidation of graphite in H_3_PO_4_ solutions resulted in the formation of the corroded carbon layers on the electrode surface [18].

Hathcock et al. [16] proposed a mechanism of blister formation, which involves gas evolution under the graphite surface at active sites where the carbon matrix oxidation takes place.

**Figure 5:**
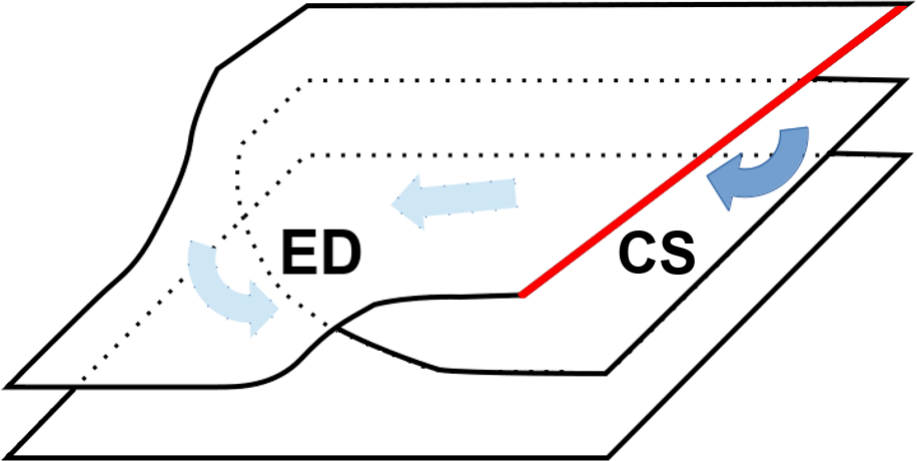
Scheme of the penetration of the intercalating agents inside the graphite through an atomic step on the surface (red line, CS) and an edge dislocation (ED) with the Burgers vector [0002] under the top layer.

We did not find any etch pits or microcracks on the HAPG surface after the treatment. Grain boundaries within the ordered regions have a well-organized structure [19], and, in our assumption, they do not permit the diffusion of the reagents inside the material. Line-shaped blisters were found along the grain boundaries (Figure S3, Supporting Information File 1) after the treatment. This may indicate that defects in the sp^2^-lattice promote the blister development. We suppose that the intercalation of the HAPG proceeds through the most frequently occurring defects: the cleavage steps on the surface and the edge and screw dislocations inside the material, as illustrated in Figure 5. The proposed mechanism may significantly influence the kinetics of pyrolytic graphite intercalation. Since molecules and ions diffuse through the system of pre-existing defects, the intercalation process can be reversible. The experimental data obtained in [20] by Raman spectroscopy proved that concentrated sulfuric acid could electrochemically intercalate HOPG reversibly without the destruction of the sp^2^-lattice.

**Figure 6:**
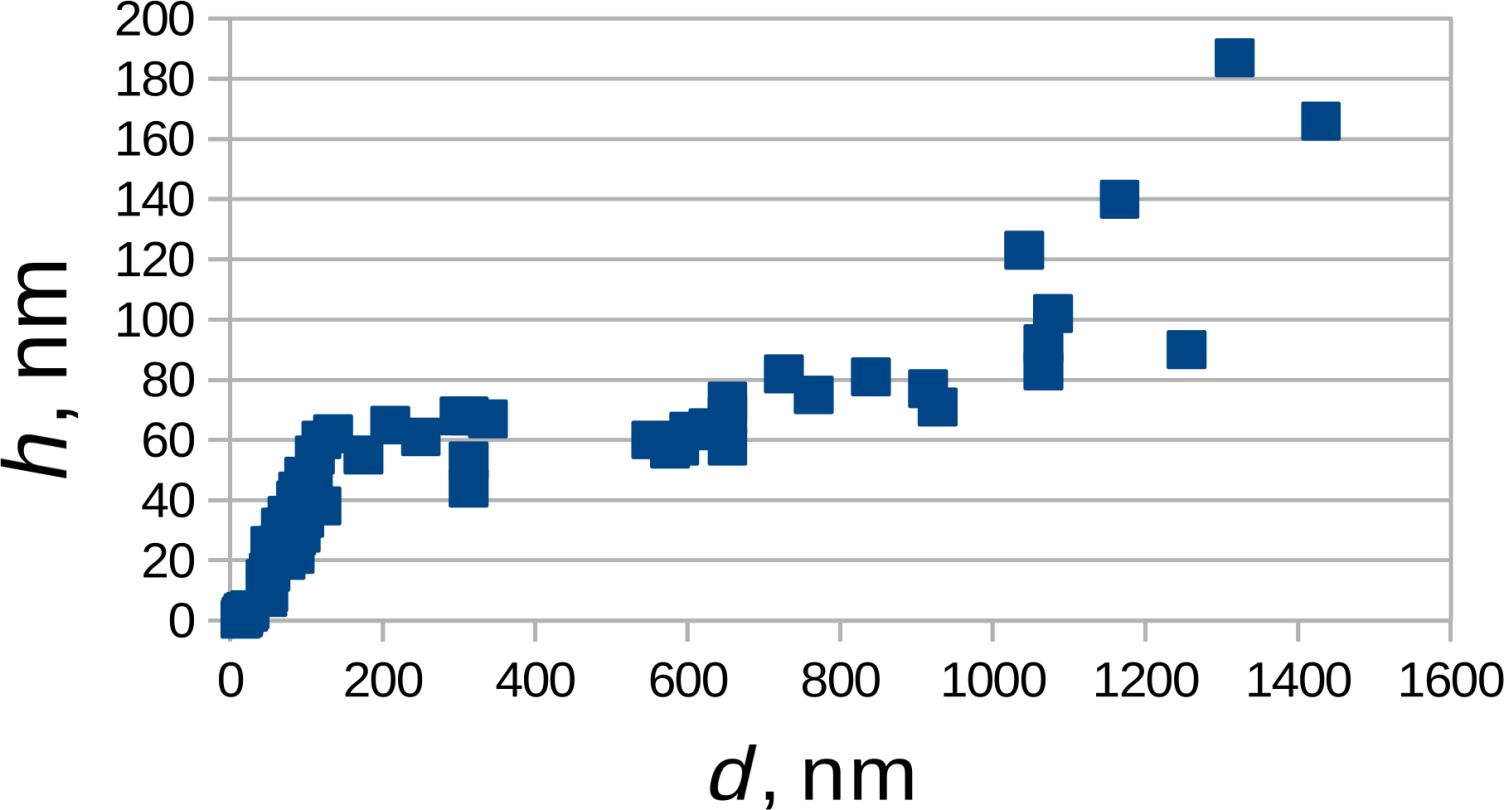
Height (*h*) vs diameter (*d*) of the blisters.

The dependence of the blister height on their diameter, shown in Figure 6, is rather complex. The curve is linear from 0 to 120 nm. With a further increase in diameter, the height of the blisters varies insignificantly from 60 to 80 nm. The blisters with a diameter of more than 1000 nm have a wide height distribution (up to 190 nm). As an example, the profiles for the large and small blisters are shown in Figure S4, Supporting Information File 1. In [16], two types of blisters were found. Small ones had a height of 30–200 nm and a diameter of up to one micrometer. The height of large blisters was hundreds of nanometers with a diameter of 1–50 μm. Similar structures, i.e., graphene bubbles, were obtained in [21]. There, a pressure difference of 0.21 MPa was applied to create a bubble with a height of 184 nm and a diameter of several micrometers. In the present study, an increase in the surface area of 6–7% was observed due to the blister formation, which would require a significant increase of the pressure in the interlayer space. However, if the carbon layers were not oxidized, according to the Raman spectroscopy data, gaseous products should not be released under the surface as in the model of Murray [15–16]. We assume that the initially formed GIC with sulfuric acid was swollen with the blister formation due to water uptake. Taken this into account we can explain Figure 6. The larger the height and the smaller the diameter, the greater the pressure in the blister should be [21]. But the shape of small blisters is almost a hemisphere, which minimizes the ratio of the surface area to volume for a nucleated liquid phase. When the diameter reaches a certain critical value of ≈120 nm, blisters begin to grow (mostly in width).

**Figure 7:**
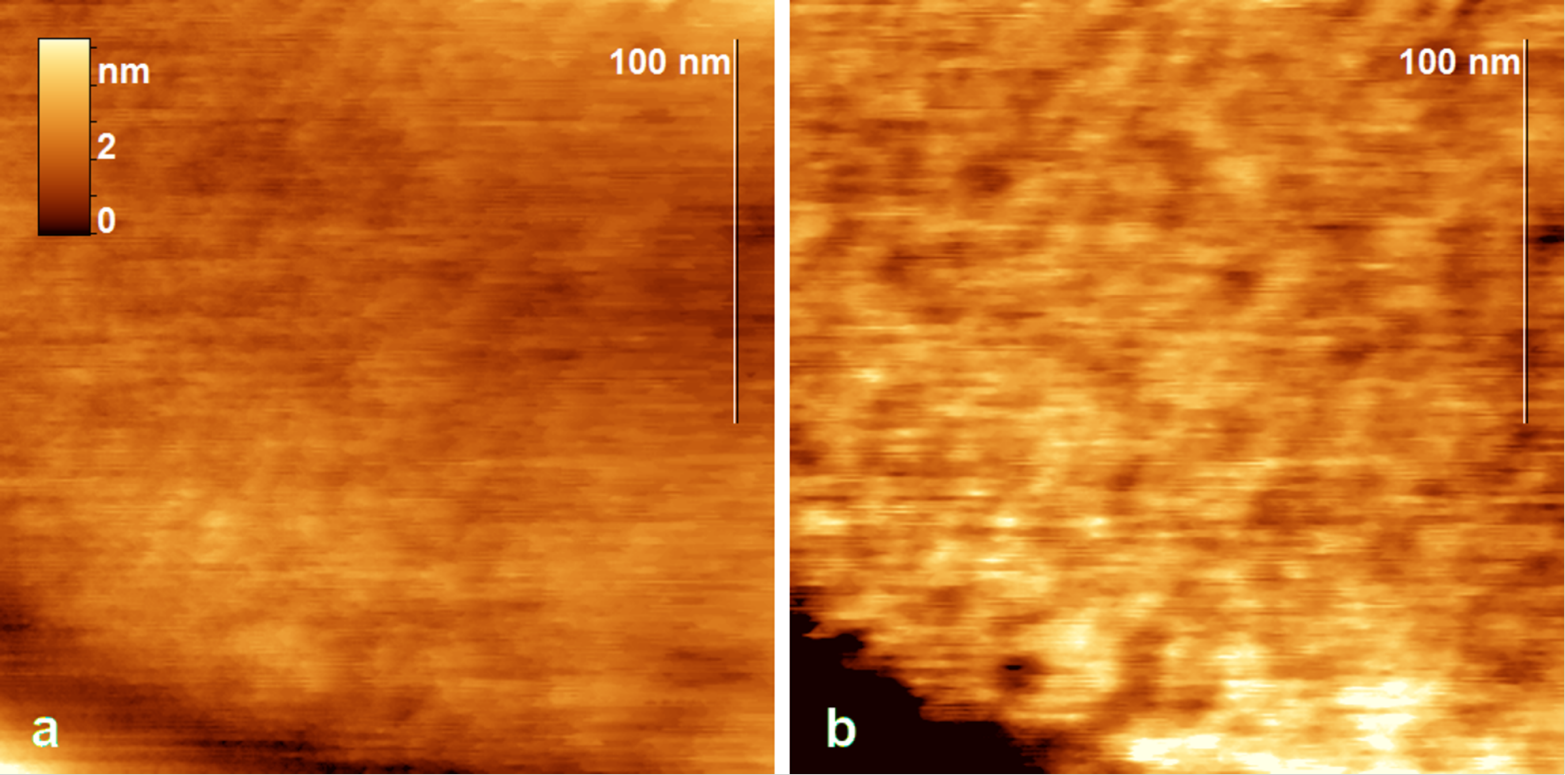
Height (a) and phase (b) images of the surface of a huge blister (the diameter is 500 nm and the height is 48 nm). The blister is shown in Figure S4, Supporting Information File 1.

A bumpy morphology was revealed on the surface of large blisters (Figure 7a,b), which is especially evident in a phase image (Figure 7b). The characteristic diameter of the bumps was 10 nm and their height is about 0.5 nm. This morphology could result from the heterogeneous distribution of sulfuric acid in subsurface layers of the residual GIC, whose presence was confirmed by Raman spectroscopy. Thus, the distance between the layers in graphite bisulfate is 0.798 nm [22], which is 0.463 nm larger than in graphite (0.33538 nm [22]). It is interesting to note that a similar surface relief was observed in [23] for graphene oxide obtained by Hummers’ method. Most likely, graphene oxide inherits the microstructure from an intermediate GIC.

**Figure 8:**
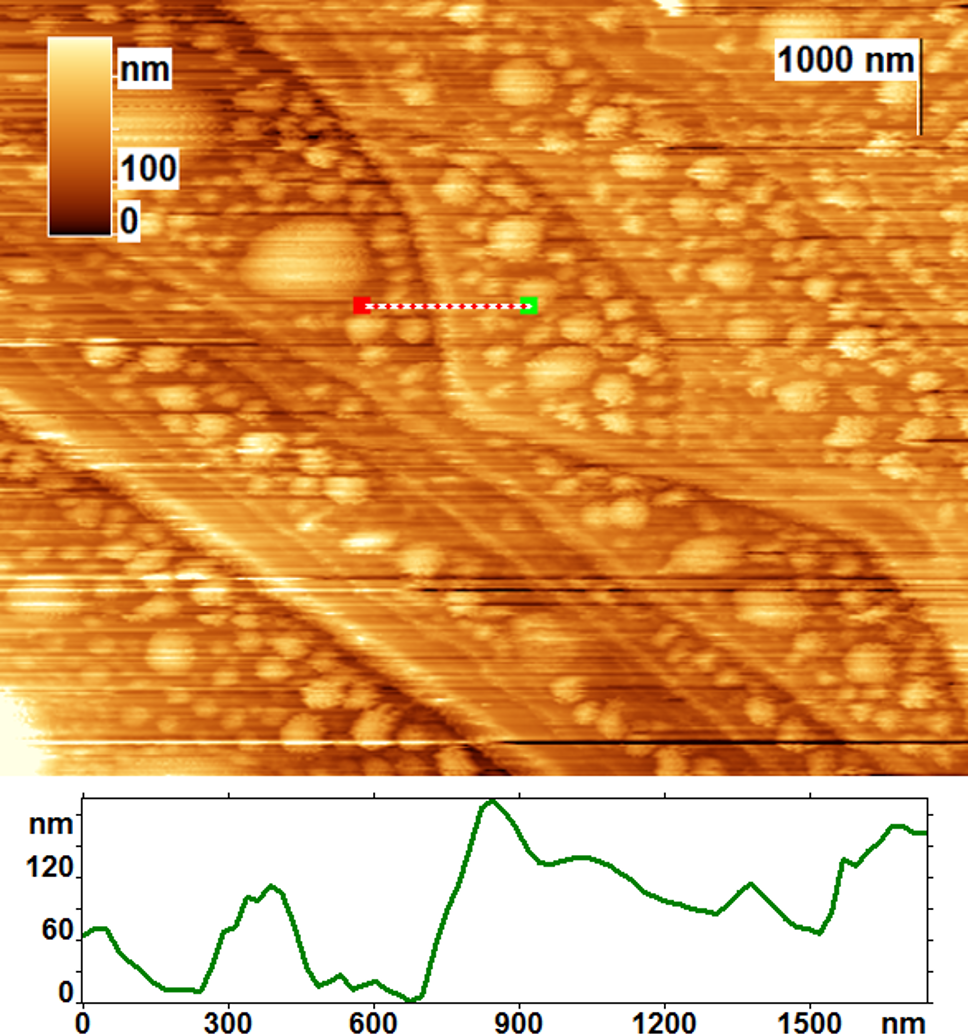
AFM image of a bunch of the cleavage steps on a highly annealed pyrolythic graphite (HAPG) surface and a line profile, which was taken from a dotted line marked in the image.

Particular attention was paid to regions containing bunches of the cleavage steps (Figure 8). Some steps became wavy, probably due to the etching by the oxidation mixture. We found that the blister density becomes larger before the large cleavage steps (more than 10 nm). We believe that the blisters cannot expand under a rather thick graphite layer because of its high rigidity. The blisters did not form only near the edges of the atomic terraces. The width of the depleted zones is from 180 nm to 600 nm. Thus, the cleavage steps strongly influence the arrangement of the blisters and can restrict their maximum size. The height of the surface near the cleavage edges increased by 20–100 nm. Perhaps the expansion of graphite at the step edges was due to the formation of GO. These data are in agreement with previous observations [6] that GO begins to form from the edges of graphite flakes.

## Conclusion

The present work showed that the short-term treatment of the basal-plane surface of graphite by Hummers’ method resulted in the formation of the blisters and the high-stage GICs with sulfuric acid (stage >2) without destruction of sp^2^-carbon lattice. We proposed a mechanism of the reagent penetration inside the graphite through the cleavage steps and the edge and screw dislocations with the Burgers vector parallel to the *c*-axis. We believe that the obtained data are important for understanding the influence of the graphite precursor microstructure on the synthesis processes of GICs and GO, and the morphology of the resulting materials.

The present research was focused on the effects of short-term oxidation. The microscopic study of long-term oxidation may be an intriguing task for future investigations. Also the study of the blister formation on the graphite samples with different microstructure is of considerable interest.

## Experimental

### Chemical treatment of the surface of pyrolytic graphite

HAPG was provided by Optigraph GmbH (Germany). Samples of HAPG with a size of ≈10 × 10 mm^2^ were cleaved before the experiment. Then, an oxidation mixture was prepared consisting of 5 mg of sodium nitrate, 30 mg of potassium permanganate, and 230 μL of concentrated sulfuric acid (obtained from Sigma Tec). 10 μL of the mixture was applied to the basal-plane surface of the HAPG. After 30 minutes, the samples were washed in a stream of milli-Q water (total volume of about 1 mL), in 3% hydrogen peroxide until the discoloration of the surface, and again in water. The samples were dried in air within a day.

An additional experiment was conducted in which the time of the treatment with the oxidation mixture was 3 minutes.

### Sample characterization

AFM measurements were carried out using a FemtoScan multifunctional scanning probe microscope produced by the Advanced Technologies Center. The surface topography was studied in a semi-contact mode in air at room temperature. We used Etalon HR11 cantilevers with average resonant frequencies of 380 kHz and 230 kHz with a tip radius of about 10 nm.

The AFM data were processed and analyzed in the FemtoScan online software [24]. The length of the cleavage steps per square micrometer was measured with the help of the curve selection tool. The measurements were carried out using ten AFM images with a size of 10 × 10 μm^2^. The sum of the step lengths was calculated for each image, then it was divided by the area of the image. The length of the dislocation lines per square micrometer was measured in a similar way. The size of the blisters was measured manually using line profiles from eight AFM images with different sizes (from 1 × 1 μm^2^ to 20 × 20 μm^2^). A total of 80 blisters were analyzed.

A Supra 50 VP Leo scanning electron microscope was used for the HAPG morphology study. The samples were tilted relative to the electron beam by 45°, which enhances the contrast between the crystallites [25].

A Renishaw InVia Raman spectrometer was used. The spectra were recorded using a 514.4 nm laser with a power of 14 mW.

## Supporting Information

File 1 Additional atomic force microscopy results.
