# Supplementary material for: Blister formation during graphite surface oxidation by Hummers’ method
Source: Beilstein J Nanotechnol. 2018 Feb 2;9:407–14. doi: 10.3762/bjnano.9.40 (PMC5815287; doi:10.3762/bjnano.9.40)
Supplement: File 1 — Additional atomic force microscopy results. [file Beilstein_J_Nanotechnol-09-407-s001.pdf]

Supporting Information

for

Blister formation during graphite surface oxidation by Hummers' method

Olga V. Sinitsyna*[1], Georgy B. Meshkov[2], Anastasija V. Grigorieva[3], Alexander A. Antonov[4], Inna G. Grigorieva[4], and Igor V. Yaminsky[1,2]

Address: [1]Laboratory for Physical Chemistry of Polymers, A. N. Nesmeyanov Institute of Organoelement Compounds of Russian Academy of Sciences, Vavilova St. 28, Moscow 119991, Russia, [2]Physics Department, Lomonosov Moscow State University, Leninskie Gory, Moscow 119991, Russia, [3]Department of Materials Science, Lomonosov Moscow State University, Leninskie Gory, Moscow 119991, Russia and [4]Optigraph GmbH, Rudower Chaussee 29, 12489 Berlin, Germany

Email: Olga Valentinovna Sinitsyna - sinitsyna@gmail.com

* Corresponding author

Additional atomic force microscopy results

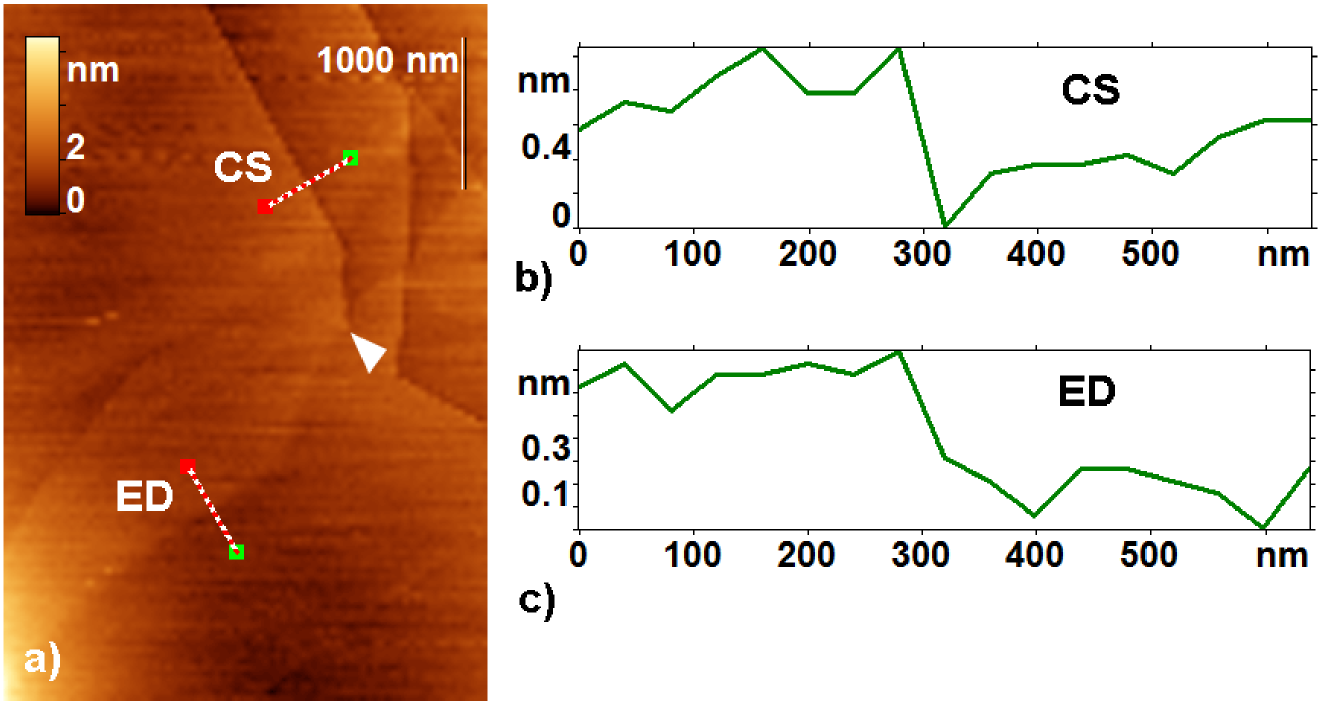

Figure S1: a) AFM image of a screw dislocation with the Burgers vector perpendicular to the basal plane. The location of the screw dislocation on the HAPG surface is marked by a white arrow. An edge dislocation (ED) and a cleavage step (CS) start from this point. Line profiles of the cleavage step and the edge dislocation are shown in b) and c), respectively.

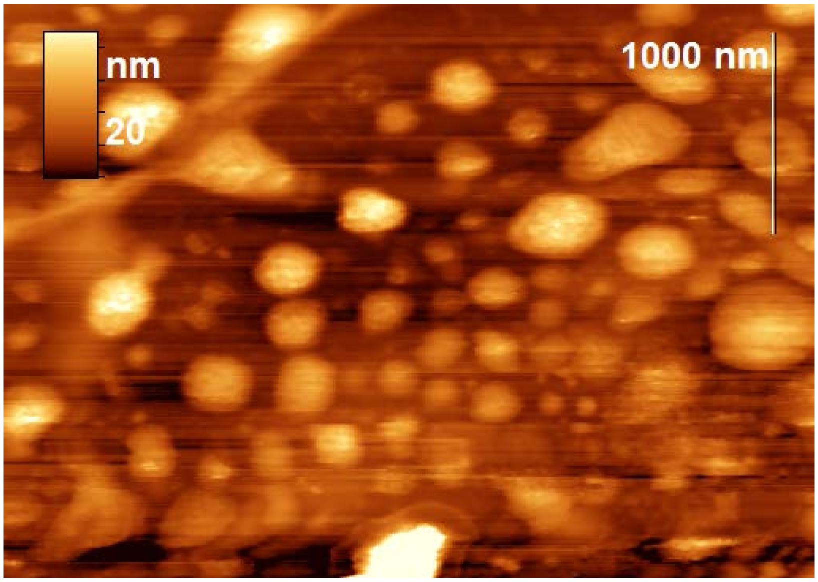

Figure S2: AFM image of the blisters on the HAPG surface after it was treated with the oxidation mixture for 3 minutes.

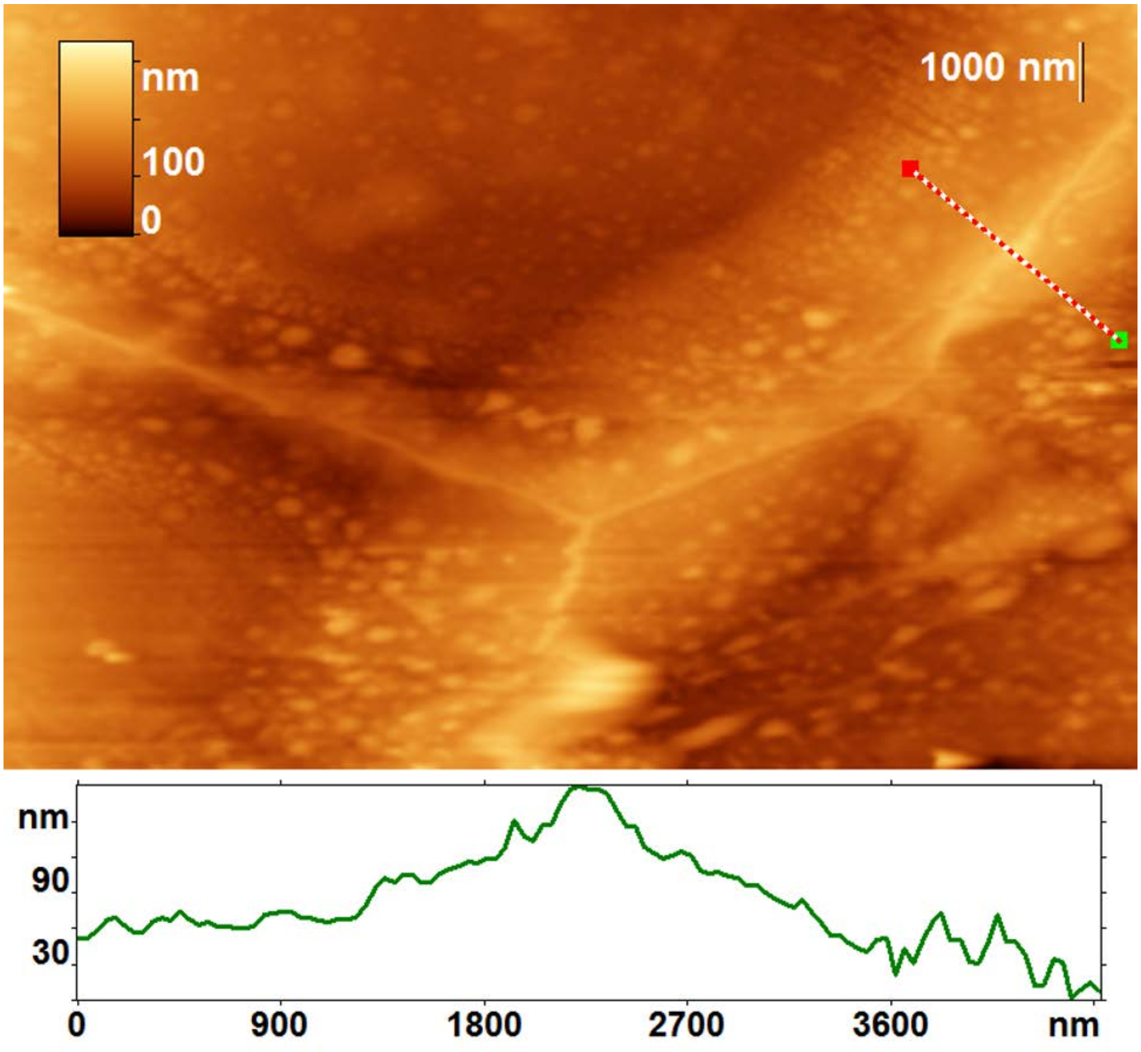

Figure S3: AFM image of grain boundaries on the HAPG surface after it was treated with the oxidation mixture for 3 minutes, and a line profile, which was taken from a dotted line marked in the image.

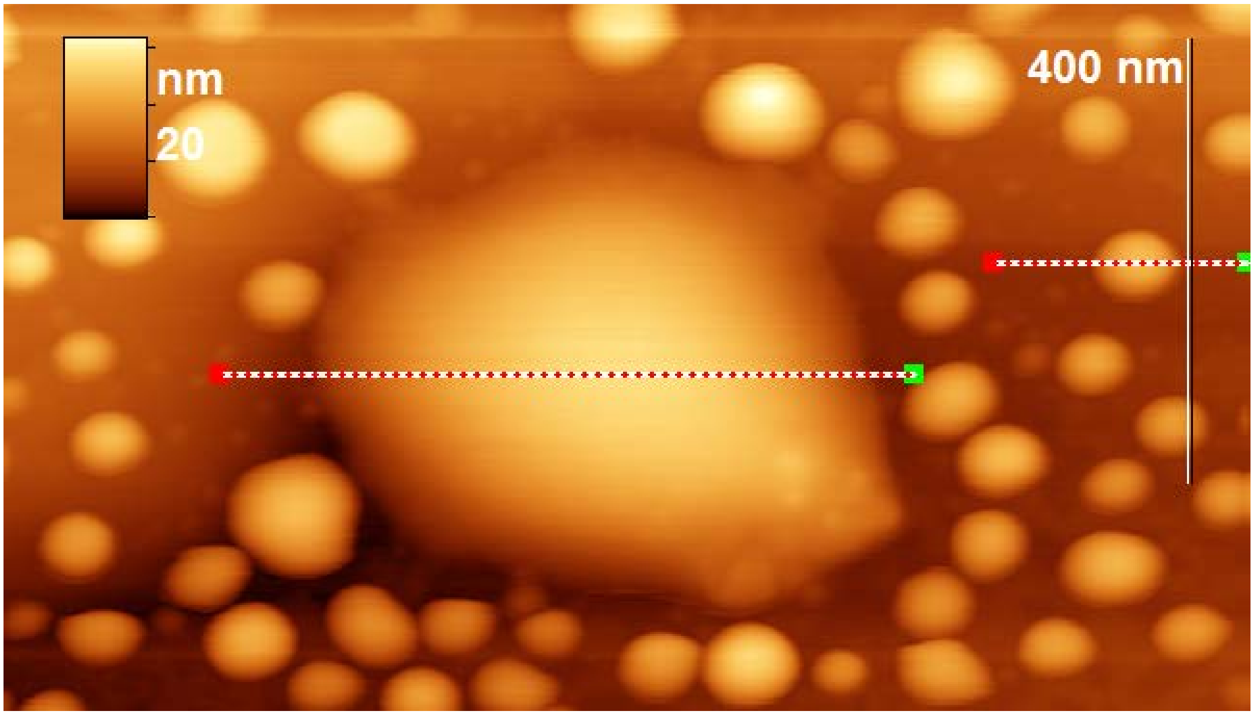

a)

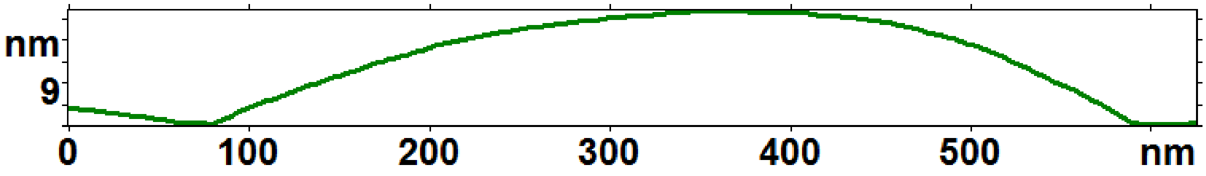

b)

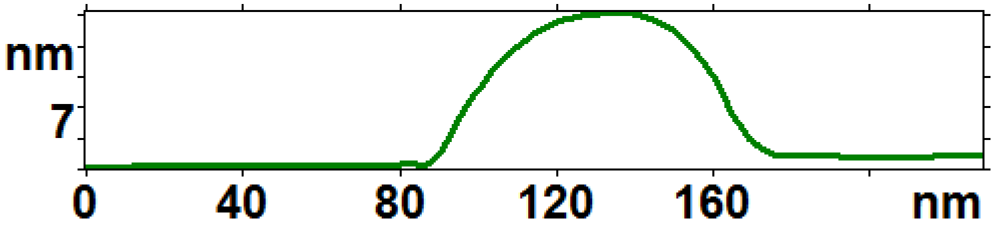

c)

Figure S4: a) AFM image of the blisters on the HAPG surface after it was treated with the oxidation mixture for 30 minutes. The profiles of large (the diameter is 500 nm and the height is 48 nm) and small (the diameter is 80 nm and the height is 35 nm) blisters are shown in b) and c), respectively.
